# Citrus green fruit detection *via* improved feature network extraction

**DOI:** 10.3389/fpls.2022.946154

**Published:** 2022-11-30

**Authors:** Jianqiang Lu, Ruifan Yang, Chaoran Yu, Jiahan Lin, Wadi Chen, Haiwei Wu, Xin Chen, Yubin Lan, Weixing Wang

**Affiliations:** ^1^ College of Electronic Engineering (College of Artificial Intelligence), South China Agricultural University, Guangzhou, China; ^2^ Guangdong Laboratory for Lingnan Modern Agriculture, Guangzhou, China; ^3^ National Center for International Collaboration Research on Precision Agricultural Aviation Pesticide Spraying Technology, Guangzhou, China; ^4^ Vegetable Research Institute, Guangdong Academy of Agricultural Sciences, Guangzhou, China; ^5^ Guangdong Key Laboratory for New Technology Research of Vegetables, Guangzhou, China; ^6^ Guangdong Provincial Agricultural Information Monitoring Engineering Technology Research Center, Guangzhou, China

**Keywords:** instance segmentation, Mask-RCNN, feature fusion, CB-Net, deep learning

## Abstract

**Introduction:**

It is crucial to accurately determine the green fruit stage of citrus and formulate detailed fruit conservation and flower thinning plans to increase the yield of citrus. However, the color of citrus green fruits is similar to the background, which results in poor segmentation accuracy. At present, when deep learning and other technologies are applied in agriculture for crop yield estimation and picking tasks, the accuracy of recognition reaches 88%, and the area enclosed by the PR curve and the coordinate axis reaches 0.95, which basically meets the application requirements.To solve these problems, this study proposes a citrus green fruit detection method that is based on improved Mask-RCNN (Mask–Region Convolutional Neural Network) feature network extraction.

**Methods:**

First, the backbone networks are able to integrate low, medium and high level features and then perform end-to-end classification. They have excellent feature extraction capability for image classification tasks. Deep and shallow feature fusion is used to fuse the ResNet(Residual network) in the Mask-RCNN network. This strategy involves assembling multiple identical backbones using composite connections between adjacent backbones to form a more powerful backbone. This is helpful for increasing the amount of feature information that is extracted at each stage in the backbone network. Second, in neural networks, the feature map contains the feature information of the image, and the number of channels is positively related to the number of feature maps. The more channels, the more convolutional layers are needed, and the more computation is required, so a combined connection block is introduced to reduce the number of channels and improve the model accuracy. To test the method, a visual image dataset of citrus green fruits is collected and established through multisource channels such as handheld camera shooting and cloud platform acquisition. The performance of the improved citrus green fruit detection technology is compared with those of other detection methods on our dataset.

**Results:**

The results show that compared with Mask-RCNN model, the average detection accuracy of the improved Mask-RCNN model is 95.36%, increased by 1.42%, and the area surrounded by precision-recall curve and coordinate axis is 0.9673, increased by 0.3%.

**Discussion:**

This research is meaningful for reducing the effect of the image background on the detection accuracy and can provide a constructive reference for the intelligent production of citrus.

## 1 Introduction

Citrus is an important cash crop in China, with an annual production of nearly 50 million tons. Scientific planning of fruit preservation and thinning is an important measure for ensuring citrus yield. During the green fruit stage of citrus, fruit development is easily affected by the environment, pests, and diseases, which results in deformed fruit, fruit with pests and diseases, and fruit with mechanical damage. The edible value of these fruits is very low, and there is little economic benefit. However, they absorb some of the nutrients of the fruit tree during the development process, which results in a waste of nutrients such that the normal fruit cannot obtain enough nutrient supply. At the same time, there are too many fruits on adult citrus trees, and the phenomenon of nutrient competition among fruits is serious. Therefore, it is important to accurately define the green fruit stage of citrus through scientific methods and to reasonably thin the fruit to improve the yield of citrus (Yan et al., 2021). The citrus green fruit stage is traditionally judged by the fruit grower’s visual observation, which not only results in subjective judgment errors (Linker et al., 2012; He et al., 2016) but also has limitations for the intelligent and unmanned operation of orchard fruit thinning. With the development of smart agriculture applications, modern computer science and technology provide new strategies for crop target identification and detection, and real-time processing of orchard image data through sensor systems and high-performance computers (Yamamoto et al., 2014; Haseeb et al., 2020) can greatly reduce labor costs and improve detection accuracies. Therefore, it is important for growers to make orchard patrol plans according to the growth of citrus green fruit, and analyzes the pictures of citrus orchard taken by the camera using deep learning algorithm, so as to obtain the current number of citrus green fruits, so that growers can determine the yield of fruit trees and carry out timely fruit thinning operation.

Machine learning is used to accomplish the task of classification. Through supervised learning, fitting of a model to data (or a subset of data) that have been labelled– where there exists some ground truth property, which is usually experimentally measured or assigned by humans (Greener et al., 2022). Subsequently, this model is used to map all the inputs into the corresponding outputs and make a simple judgment on the outputs for prediction and classification, which also has the ability to predict andclassify the unknown data. Yoosefzadeh et al. (Yoosefzadeh Najafabadi, 2021) implemented ML algorithms in GWAS, investigated the potential use of RF and SVM algorithms in GWAS to detect the associated QTL with soybean yield components, which would be beneficial to select the superior soybean genotypes. Therefore, integrating artificial intelligence and computer vision technology to establish a citrus green fruit stage detection model would be an effective smart agriculture approach (Yang et al., 2021) for detecting citrus (Rakun et al., 2011; Lin et al., 2019).

For target detection and recognition of citrus fruits, traditional machine learning methods mainly use learning algorithms such as edge detection algorithms, watershed segmentation algorithms, and support vector machine algorithms (Kurtulmus et al., 2011; Lu and Hu, 2017; Peng et al., 2021). Such methods design feature extraction algorithms for the color, texture, and shape of crops or agricultural products (Li et al., 2017), segment relevant features in steps, and accurately locate targets in images (Wang L et al., 2022). For example, (Dorj et al., 2017) proposed a color feature-based citrus yield estimation algorithm.

Based on the automatic watershed algorithm, distance conversion and marker control methods have been introduced, which can better segment the individual citrus fruits in images. (Hu, 2018) improved machine learning-based citrus green fruit detection by using the local binary method and the maximum stable polar region algorithm to extract the color images in the region of interest, using the Hough transform to fit each level of contour lines to obtain a hierarchical circular target, and finally, performing a fitted circle nested analysis to obtain the citrus green fruit target. The above methods describe individual features of field crops in color, texture, and shape space to achieve target and background segmentation.

These traditional field crop detection methods require high background complexity of the input image, and their performance on complex and diverse agricultural orchard scenes is limited. In addition, these methods perform feature extraction on a single scale and fail to produce high-accuracy detection results. Instance segmentation algorithms provide pixel-level target detection methods for solving the problem of inaccurate classification due to individual deformation of targets of the same category in target detection methods and achieving the detection of different individuals of targets of the same category (Liu et al., 2018; Wang et al., 2020; Jia et al., 2021). The most widely used instance segmentation algorithm is the Mask-RCNN (He et al., 2017) algorithm (Wang et al., 2016), which applies the extended convolution method to the Res4b module of ResNet, which is the backbone network of Mask-RCNN, for the recognition and localization of poplar plum in the natural environment to achieve accurate recognition and segmentation of poplar plum. Zhang Y et al. (Zhang, 2020) used the Mask-RCNN algorithm with a Kinect V2 (Lachat et al., 2015) camera to acquire apple images under different environmental conditions and to segment the generated apple point cloud data (Wahabzada et al., 2015). Deng Y et al. (Deng et al., 2020) achieved efficient detection of dense small-scale citrus flower targets in complex structured images and acquired the number of visible flowers in images by optimizing the body convolution part and the mask branching part of the Mask-RCNN algorithm. The Mask-RCNN algorithm has high-efficiency detection performance and high operability and is widely used in various field crop detection and segmentation (Santos et al., 2020) tasks. It is an important tool for implementing instance segmentation tasks in agriculture.

Current research has focused on target recognition of ripe citrus yellow fruits, and less research has been conducted on the detection of citrus green fruits (Zhao et al., 2016), but it is important to accurately identify citrus green fruits and define the citrus green fruit stage. Compared with ripe citrus yellow fruits, citrus green fruits are more difficult to recognize. The reasons are as follows: (1) citrus green fruits are difficult to distinguish because their color is similar to the background under natural light (2) under natural conditions, citrus green fruits are small in size and occupy very few pixels in the image (3) citrus green fruits overlap each other and thus are easily blocked by leaves, branches and other background objects, which is difficult to detect. Therefore, we need to more accurately extract the inherent characteristics of citrus green fruits, fuse the multiclass features of citrus green fruit, and use efficient feature extraction methods to improve the accuracy of citrusgreen fruit detection and segmentation.

Deep convolutional neural networks are constantly evolving, and many backbone networks are able to integrate low, medium and high level features and then perform end-to-end classification. They have excellent feature extraction capability for image classification tasks, and common backbone networks are VGG, Resnet, etc. In neural networks, each channel needs to do convolution operation with a convolution kernel, and then the results are summed to get a feature map output. The feature map contains the feature information of the image, and the number of channels is positively related to the number of feature maps. The more channels, the more convolutional layers are needed, and the more computation is required, so reducing the number of channels is beneficial to the computation speed.

This study selects citrus green fruits in the natural environment as the research objects due to the limited accuracy of traditional target detection algorithms for detecting citrus green fruits in complex backgrounds. Based on an improved version of the pixel-level instance segmentation algorithm Mask-RCNN (Fan et al., 2021), a citrus green fruit detection method is designed by introducing CB-Net (Composite Backbone Network) (Liu et al., 2020). The method involves assembling multiple identical backbones using composite connections between adjacent backbones to form a more powerful backbone. This helps increase the feature information that is extracted at each stage in the backbone network. Then, a combined connection block is introduced to reduce the number of channels and improve the model accuracy. This method can effectively mitigate the problem that the citrus green fruit color is similar to the background color, which reduces the detection accuracy. The proposed algorithm is pretrained by combining the data of citrus green fruit images that were captured from multiple angles using a camera and cloud platform with the training weight file of the Mask-RCNN algorithm on the COCO dataset. Then, the algorithm is formally trained and tested on the collected citrus green fruit images for evaluation. This study provides a research basis for the tasks of detecting and dividing citrus green fruits under natural conditions and the development of intelligent and unmanned operations for citrus green fruit thinning, and it broadens the research scope of intelligent agriculture in the field of citrus flower and fruit preservation. It is important to improve the efficiency of citrus operations and promote the development of citrus production.

In this study, the image data of citrus green fruit under real natural environment was collected, and the corresponding data enhancement processing was carried out to construct a citrus green fruit data set. Based on the Mask-RCNN algorithm, CB-Net with deep and shallow fusion was innovatively combined with the traditional feature extraction network ResNet to fuse the multilayer features of citrus green fruits. Then, the proposed model was compared with the traditional model in detail. Theresults showed that the proposed algorithm based on the improved Mask-RCNN has improved citrus green fruit detection accuracy and speed.

The main contributions of this study are as follows:

(1). Based on the strategy of feature fusion, an instance segmentation method is proposed for reducing the influence between citrus green fruit features and irrelevant features.

(2). We construct a novel Mask-RCNN model using CB-Net (a composite backbone network) to fuse the multilayer features of citrus green fruits so that the model can focus more on the obvious regions and more detailed features in each image.

(3). The performance of the proposed model in detecting different individuals under the same class of target to obtain the morphology of citrus green fruits in advance is evaluated.

## 2 Materials and methods

### 2.1 Study area

**Figure 1:**
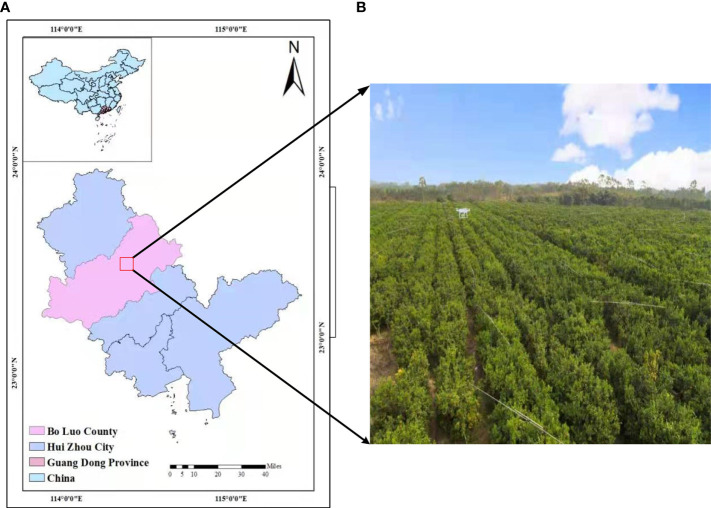
Study Area: **(A)** Geographical location of the study area and **(B)** the citrus test base.

Sugar tangerine is an important variety in China’s citrus industry. Its main production area is South China, and it has high edible value. Its growth stage is similar to that of ordinary citrus. The cultivation of sugar tangerine has high environmental requirements, and sugar tangerine has poor resistance to insects and diseases. It is necessary to observe the growth of each tree for a long time to supplement nutrients in time and promote the normal development of flowers, fruits, and leaves (Rahnemoonfar and Sheppard, 2017). Using computer vision to detect citrus green fruit targets requires high-resolution images and imaging data to study the important shape features of citrus green fruit and to finally evaluate the accuracy of the results. The experimental site for this study is located in the research and development demonstration base for Green Plant Protection of Citrus HLB (Huanglongbing) and New Cultivation Modes in Jingshuilong Village, Yangcun Town, Boluo County, Huizhou City, Guangdong Province (N23°29’57.81”—N23°29’59.31”,E114°28’8.39”—E114°28’12.26”). It is 40 m above sea level, and the local climate is mild and humid, which is suitable for the planting of citrus and other fruit trees. The crop varieties in this test area are all sugar tangerines. There are 334 citrus plants in the test area, with 9 rows, a row spacing of 4 m, and a column spacing of 2.5 m. In the natural light environment, visible image data of citrus green fruits were collected in July 2020. The citrus experimental site is shown in **Figure 1**.

### 2.2 Test data

#### 2.2.1 Test data collection

**Figure 2:**
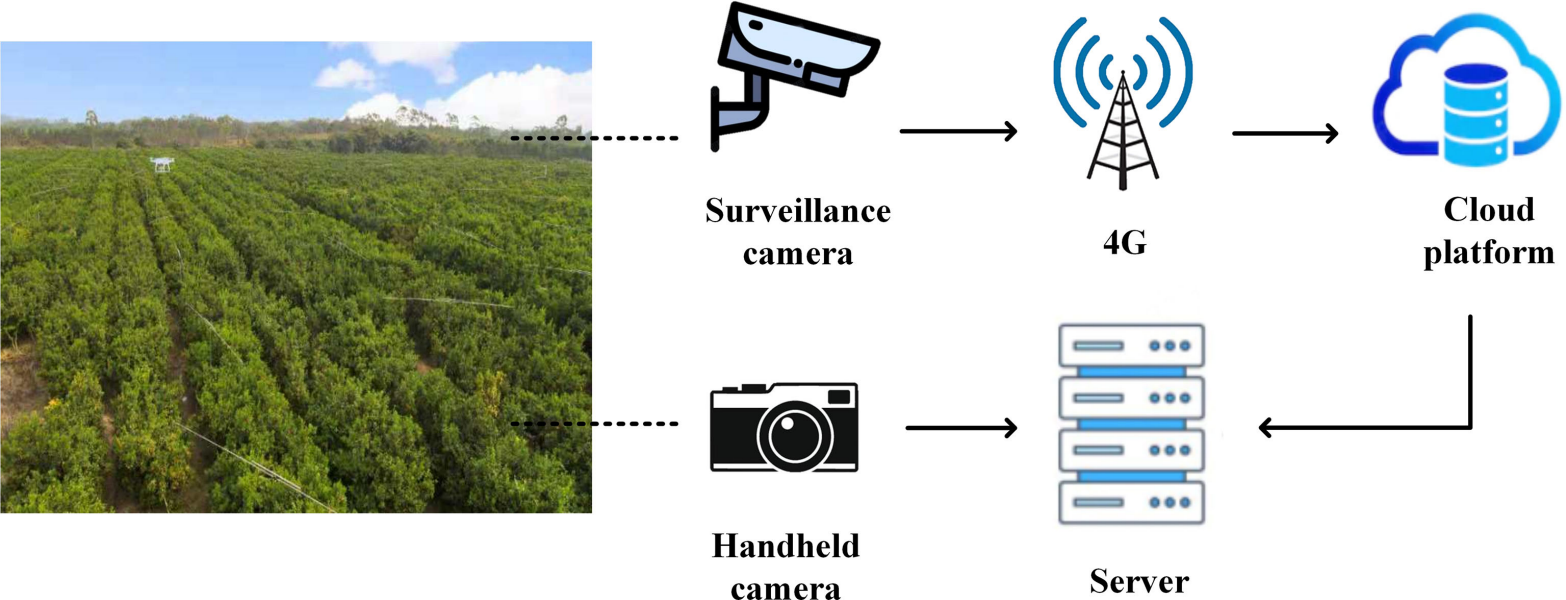
Data acquisition mode diagram.

**Figure 3:**
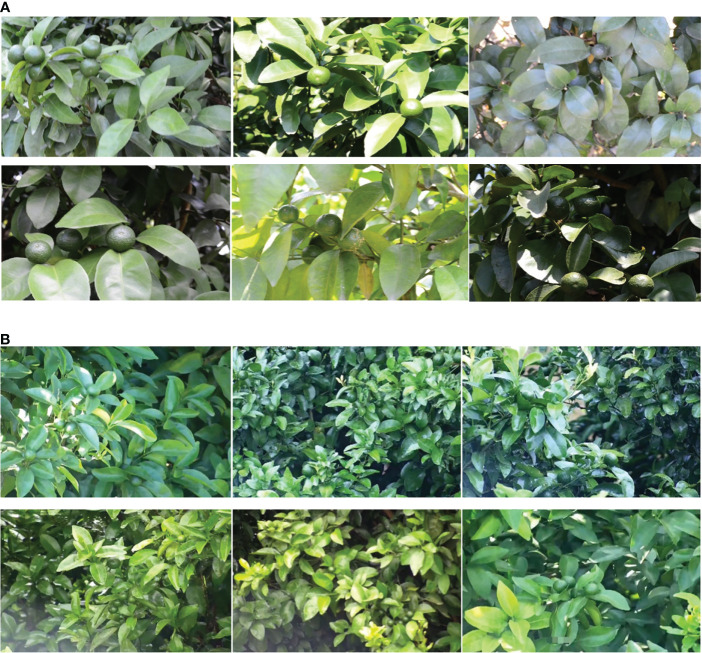
Citrus green fruit images. **(A)** Pictures of citrus green fruit that were captured with a manual handheld camera and **(B)** pictures of citrus green fruit that were obtained by the cloud platform.

To improve the quality and diversity of citrus green fruit images, the modes of the collection were as follows: 1. A manual hand-held camera (Model D7100, APS-C frame camera of Nikon, Japan) was used to take multiangle visible images of citrus green fruits at a distance of 2-3 m from the canopy of the citrus tree, which produced JPEG images with a resolution of 4928×3264; 2. A wireless zoom camera (Hikvision 3T27EWD) was called in the orchard through the cloud platform. This zoom camera has a 1/2.7 largetarget sensor. At a distance of 5-10 m from the canopy of the citrus tree, we used it for remote real-time acquisition of visible images of citrus green fruits, which produced JPEG images with a resolution of 1280×720. The data acquisition mode diagram is shown in **Figure 2**. Finally, 200 and 357 citrus green fruit images were collected using the hand-held camera and cloud platform, respectively, for a total of 557 images. The number of citrus green fruits in each image was 1-10. To obtain a unified data format, the picture resolution was compressed to 1280×720. **Figure 3** shows citrus green fruit picture data that were collected by the two data collection modes.

#### 2.2.2 Data processing

**Figure 4:**
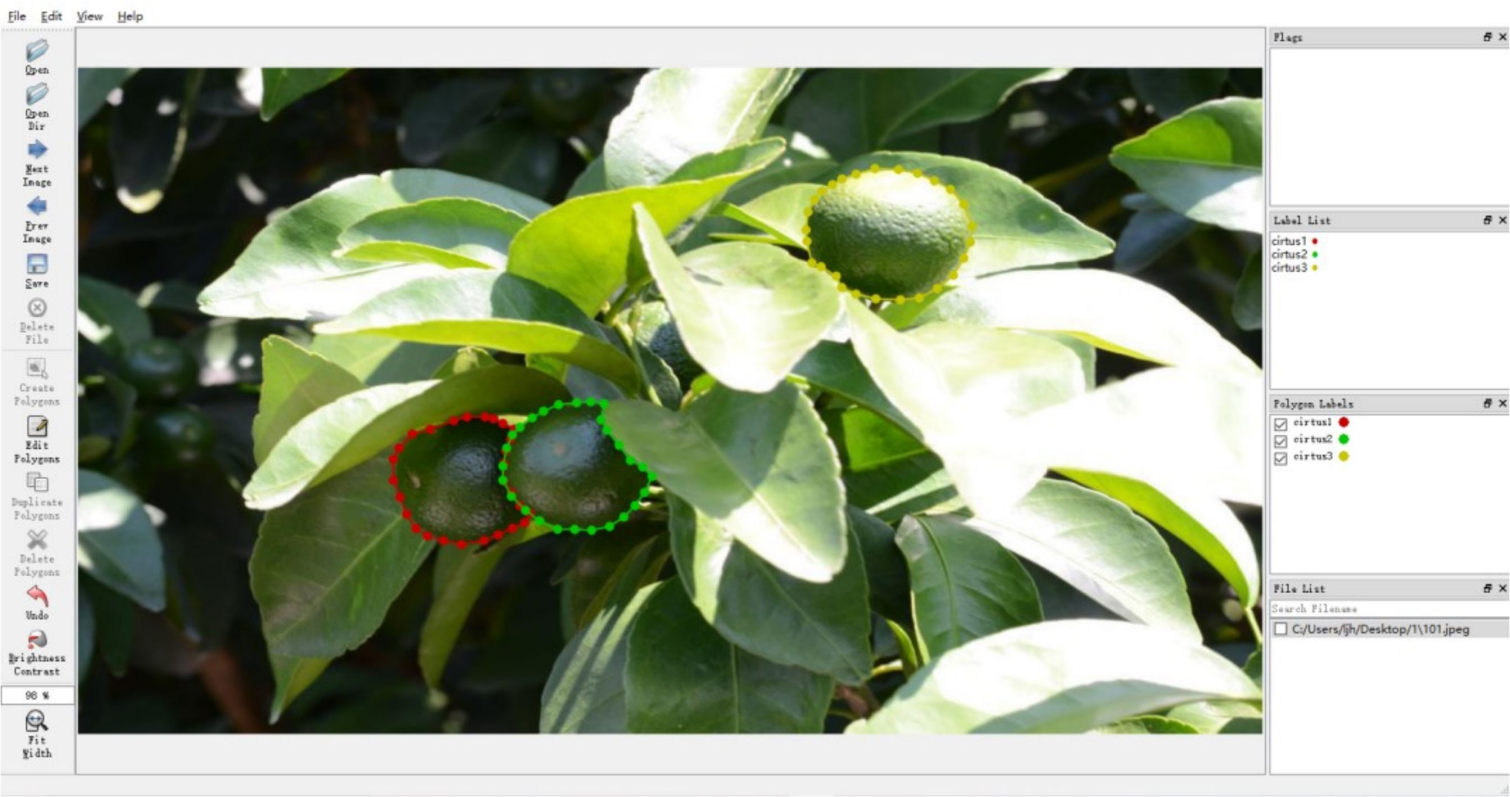
Data annotation interface.

The data in supervised learning needs to be classified in advance, and its training samples contain both feature and label information. Therefore, a method based on improved Mask-RCNN for constructing citrus green fruit dataset is proposed. First, Labelme (Russell et al., 2008) data labeling software is used for instance labeling of citrus green fruit individuals, and the interface of the data labeling software is shown in **Figure 4**. The top of the software is the menu bar, the left side is thetoolbar, including open file or folder, select the current picture before and after the picture file, save the annotation file, select the annotation method, etc., a picture annotation area in the middle, and the right side is the picture and the annotation name, category and other information. When labeling, select polygon labeling method to manually mark the citrus target with dense dotting, and mark the visible citrus target area.

The citrus green fruit of each monomer is regarded as a class. When there are multiple citrus targets in the **Figure 4**, the labels are set to “cirtus1”, “cirtus2”, “cirtus3”, and so on. When the labeling of an image is completed, a json label filewith the suffix “.json” is generated, which records the version number of Labelme software, the label of each citrus green fruit labeled and the pixel coordinates of the corresponding labeling point.

In this study, we fully consider the various shapes in the environment in which the citrus green fruits are located in the sample annotation process and ignore the citrus targets that are obscured by more than 70%. The method can accurately obtain the citrus green fruit target locations and reduce the interference of citrus green fruit with obscured feature information in images with complex backgrounds. A total of 3273 Citrus green fruit samples are labeled.

**Figure 5:**
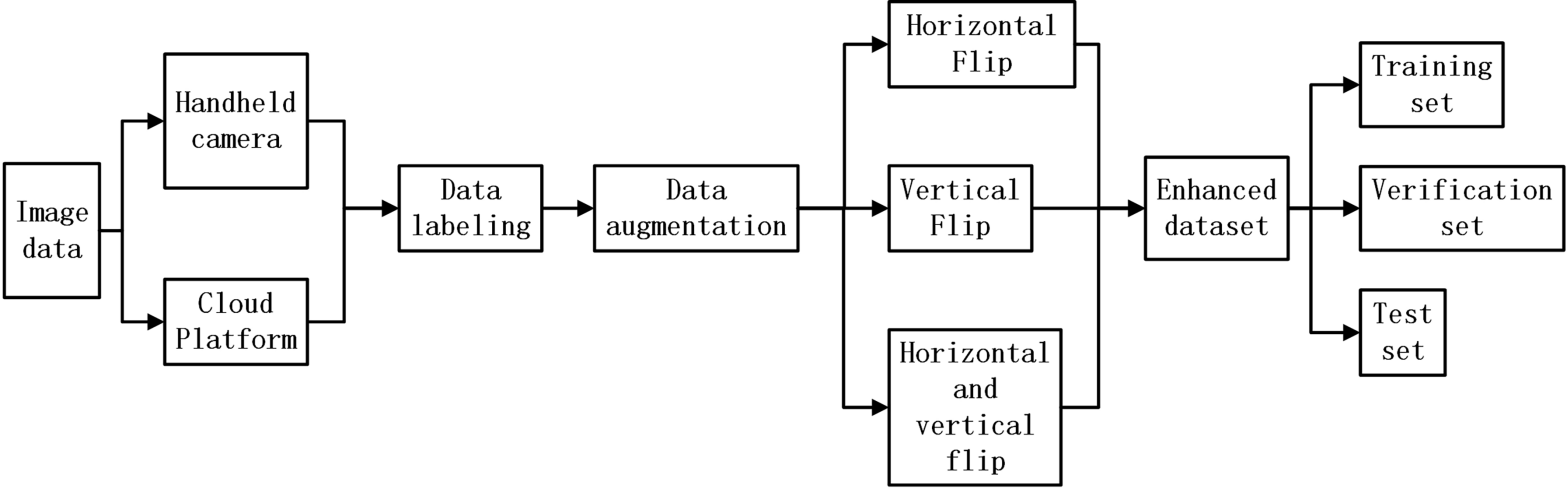
Data pre-processing flow chart.

In this study, data augmentation is used to improve the network learning and generalization ability of the network model. We mainly use image rotation, image horizontal flipping, vertical flipping, and horizontal-vertical flipping as data augmentation methods. Rotating and flipping images can improve detection performance. Meanwhile, hybrid augmentation is designed to address the limitation of the overdependence of the model on the dataset. The mixture of different classes of samples in the dataset is used to generate new samples, which enhances the linear expression among different classes of samples and improves the robustness against the samples. The size of the amplified dataset is 2228. Furthermore, the dataset is randomly divided into a training set, validation set, and test set at a ratio of 6:2:2 for training, tuning, and testing. A flow chart of the data preprocessing is shown in **Figure 5**.

### 2.3 Mask-RCNN

In deep learning, the semantic information includes the texture, color, or category of the target in the image, the richer the semantic information, the stronger the correlation between each pixel point and the surrounding pixels in the image. In the process of citrus green fruit recognition, it is necessary to preserve and integrate the feature maps with different resolutions that are generated by the feature extraction network in each convolution stage to generate feature maps with rich high-resolution semantic information.

This is especially beneficial for improving the recognition rate of green fruits and distinguishing the subtle features of fruits and leaves. In the current research, the RPN structure that is proposed by Faster-RCNN (Ren et al., 2015; Sa et al., 2016; Apolo-Apolo et al., 2020) has advantages in terms of model accuracy and training prediction speed, but traditional unidirectional networks such as ZFNet (Fu et al., 2018) and VGG16 (Qassim et al., 2018) are adopted by the CNN feature extraction network to convolve and sample the original images with high resolution and weak semantic information from top to bottom and finally generate feature maps with low resolution and strong semantic information. When the target shape or feature difference in the image is small, a low-resolution feature map will easily lose the feature information of small targets, thereby resulting in a decreased recognition rate and missed detection of the small target, among other effects.

The Mask-RCNN model is based on Faster-RCNN with the addition of a semantic segmentation branch for outputting the mask of the target and adjusting the training parameters through the loss function to achieve deep learning of image features. The Mask-RCNNalgorithm introduces a feature pyramid network into the CNN feature extraction network (Lin et al., 2017) and uses a ResNet network that is based on residual learning as the feature extraction network. In contrast, FPN in the Mask-RCNN algorithm fuses multiple feature scales and semantic information, which can realize multiscale feature extraction and fusion of images.

Mask-RCNN adds a mask prediction branch to the target detection algorithm Faster-RCNN and performs convolution and fully connected operations on the feature map in parallel with the bounding-box regression branch and classification branch. Moreover, it uses the RoI Align (Wang and He, 2019) method instead of RoI Pooling (Liu et al., 2017) of Faster-RCNN to enhance the pixel-to-pixel correspondence between network inputs and outputs, reduce the error of the bounding-box regression, and improve the target detection accuracy (Li et al., 2020). Based on the above features, Mask-RCNN enhances the feature information between citrus green fruit and the background in the process of detecting citrus green fruit, which is helpful for reducing the difficulty of citrus green fruit detection and segmentation. Therefore, this study explores the high-resolution optimization of feature maps in citrus green fruit detection based on the advantages of Mask-RCNN, which fuses multiple feature scales and semantic information to achieve multiscale feature extraction and fusion of images (He et al., 2020).

### 2.4 The proposed algorithm

The feature extraction network extracts the shape features of citrus green fruit by a convolution operation and builds a multilayer neural network model to realize the recognition and localization of citrus green fruit in images. However, in the actual scene, the citrus green fruit and the leaves are similar in color, and some of the leaves are also round-like in outline, which makes it difficult for the model that is built by a single feature extraction network to distinguish the feature information of the citrus green fruit and background, which increases the difficulty of detection and segmentation of citrus green fruit. Instance segmentation has both the characteristics of semantic segmentation and target detection. The region where the instances are located is identified by the target detection method, and then semantic segmentation is performed within the detection frame, and each segmentation result is output as a different instance. Since citrus green fruits differ from leaves in shape and color by subtle features, it is necessary to design a deep and shallow feature extraction network with both extraction and fusion functions in order to describe the inherent features of citrus green fruits more accurately. The improved algorithm is used to further extract phenotypic features such as shape and color of citrus green fruits under a green background. At the same time, the object detection is further refined to fuse the multiple classes of features of citrus green fruits. Then, the extracted multi-scale feature information is used to separate the detection object from the background and achieve accurate segmentation at the pixel level.

**Figure 6:**
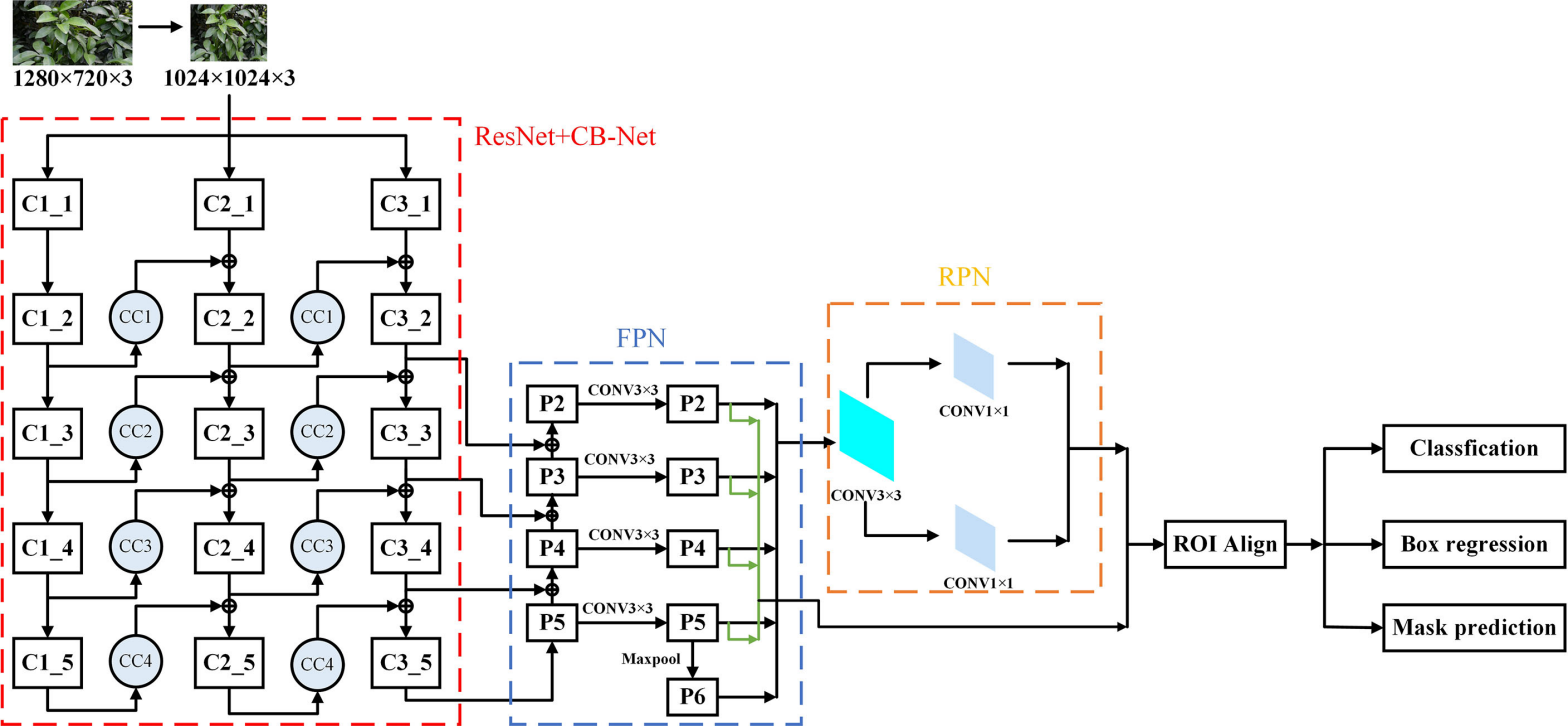
The structure of Mask-RCNN model integrating CB-Net idea.

In this study, based on the Mask-RCNN network structure, CB-Net is introduced. CB-Net provides a highly effective feature extraction method for target detection and instance segmentation algorithms based on the strategy of composite connection, which is a worthwhile optimization strategy for tasks in which detection is difficult and the feature effect is not obvious. In this study, we expect the improved algorithm to effectively identify citrus green fruits in similar background environments and obtain better model accuracy at the expense of the time cost of model training and prediction.The overall structure of the Mask-RCNN model that incorporates the CB-Net strategy is illustrated in **Figure 6**. The backbone network consists of a feature extraction network that uses the ResNet + CB-Net network and backbone, RPN, and an ROI head. The input citrus green fruit images are compressed and passed into three branches of the improved feature extraction network. ResNet based on CB-Net is used to extract and fuse multiple phenotypes of citrus green fruit, and the expression ability of multiscale features is enhanced by FPN. Furthermore, the feature maps are generated and corrected for candidates bound by the RPN and ROI align modules. Finally, three prediction branches of regression, classification (Li et al., 2019) and masking are used for the detection and segmentation of citrus green fruit. The joint loss function of Mask-RCNN is used to optimize the parameters in the model training process.

**Figure 7:**
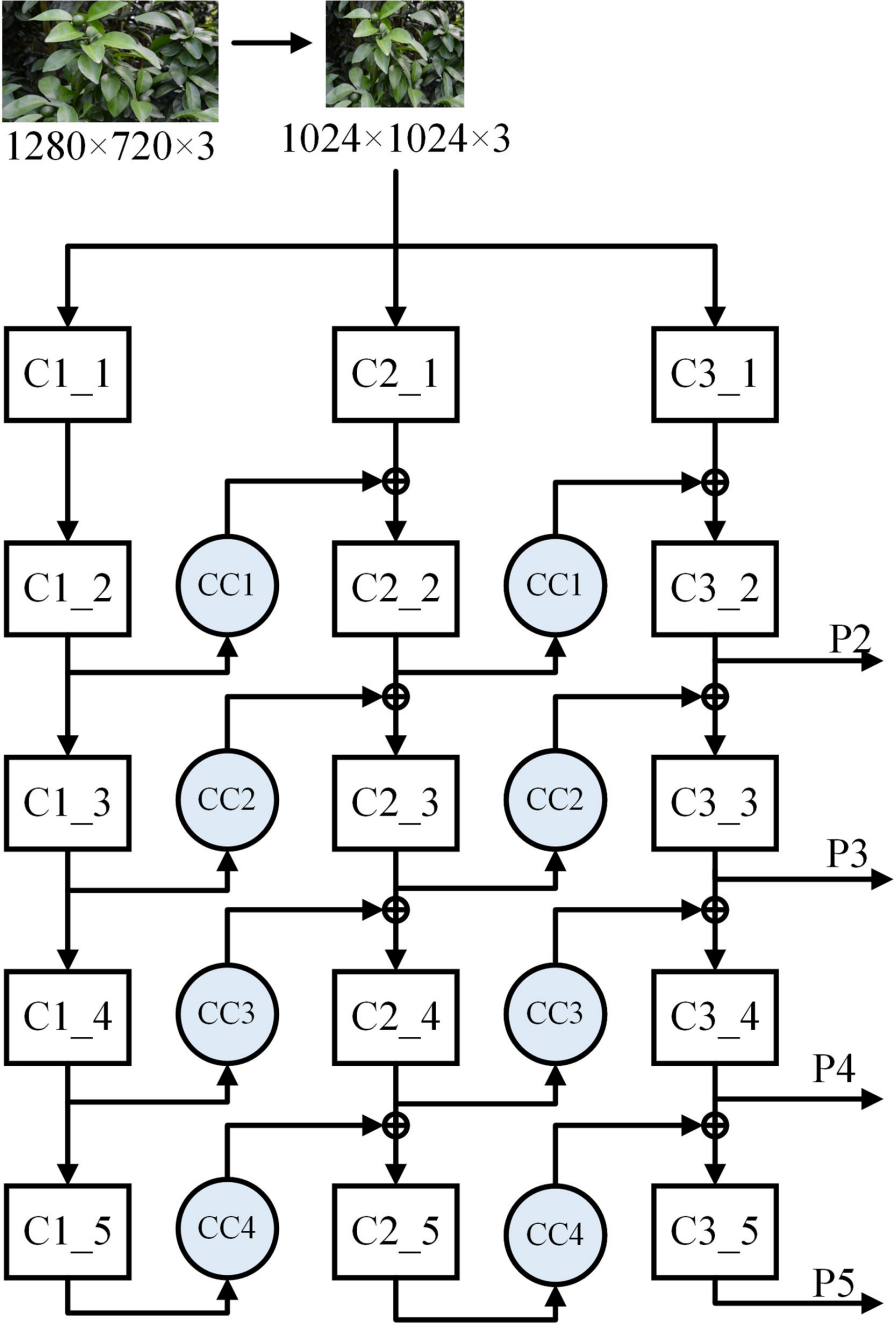
ResNet+CB-Net Network structure.

The improved Mask-RCNN uses the ResNet + CB-Net network as the feature extraction network, and the structure of the network is illustrated in **Figure 7**. The design strategy is that ResNet50 or ResNet101 (Hong et al., 2020) is iterated many times,and a composite connection module is used between each ResNet block for transverse propagation of the feature maps, which can effectively increase the amount of feature information that is extracted at each stage in the backbone network and improve the performance of citrus green fruit detection in similar background environments.

The stage names of each ResNet network are Ci-j in **Figure 7**, where i denotes the i-th ResNet network and j denotes the j-th stage. The CC module is a composite connection module, and the numbers of channels for 1×1 convolution in CC1, CC2, CC3, and CC4 are 64, 256, 512, and 1024, respectively.

A single ResNet network has fixed requirements for the size of the input images. Before images are input to the ResNet network, they need to be resized. In this study, the original images of citrus green fruit are resized to 1024×1024×3 as the first-stage input of each ResNet network. As the depth of the network increases, the size of the feature maps decreases, and the output size of stage C1 is 256×256×64, that of stage C2 is 128×128×256, that of stage C3 is 64×64×512, that of stage C4 is 32×32×1024, and that of stage C5 is 16×16×2048. An important parameter of ResNet + CB-Net is the number of network iterations. With the increase in the number of network iterations, the final extracted feature information becomes richer, and the model expression ability improves gradually. When the number of iterations reaches a threshold value, the improvement in the network accuracy decreases gradually, and as the number of iterations is further increased, the network accuracy improvement becomes close to zero. The number of iterations is linearly related to the time of model training and the capacity of the physical memory that is occupied by the hardware. When the accuracy of the network reaches the saturation condition, increasing the number of iterations has little benefit in terms of the accuracy but greatly reduces the operational efficiency of the model and increases the ratio of the storage space that is occupied by the model. To ensure that the entire network is highly efficient, the number of iterations is set to 3; that is, three identical ResNet networks are used for connections.

The network adopts AHLC mode for composite connections. The composite connection module consists of a convolutional layer with a convolutional kernel of size 1×1, a batch normalized layer (Ioffe and Szegedy, 2015), and an upsampling module. The 1×1 convolutional operation changes the number of channels. Batch normalization can improve the model efficiency and reduce regularization processing. The upsampling module changes the size of the feature image for linear matrix superposition with the convolutional layer of the next ResNet network. In one connection, we assume that the output of stage i of the first ResNet network is O1 (i). After a composite connection with C(·), O1 (i) is superimposed with the output of stage i-1 of the second ResNet network as the input I2(i) of Phase i of the second ResNet network. Then, the output O2(i) of Phase i of the second ResNet can be obtained by Formula 1, where F(·) is a convolutional operation of stage i.


(1) $$O_{2}(i)=F(I_{2}(i))=F\left(C\left(O_{1}(i)\right)\right)+O_{2}(i-1),i\geq 2$$

**Figure 8:**
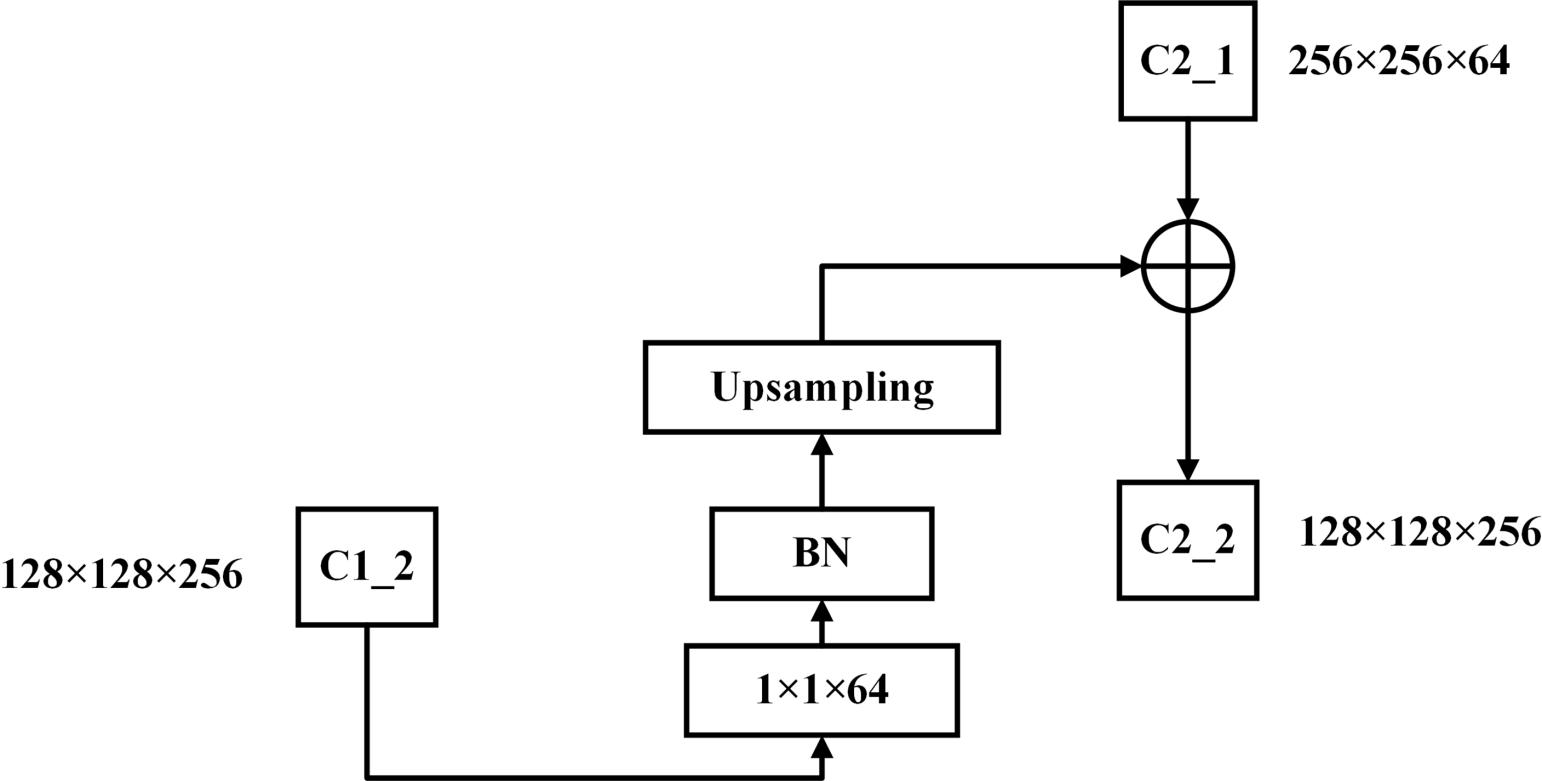
Composite connection module.

To further demonstrate the process of lateral transmission of feature information, the output of C1-2 is transmitted to the second ResNet network through a composite connection module as an example (**Figure 8**). C1-2 is the second stage of the first ResNet network, so its output feature map size is 128×128×256. The number of channels of the feature map is downsampled by CC1 with a 1×1×64 convolution, and the size of the output feature map is 128×128×64. In addition, to perform linear summation with the output feature map of C2-1, the length and width parameters of the feature map are further upsampled. Then, the size of the output feature map of CC1 is 256×256×64, which is the same as the size of the output feature map of C2-1. To generate multiple outputs from the backbone, a composite connection module is introduced. This module consists of a 1x1 convolutional layer and a batch normalization layer. These layers are added to reduce the number of channels and to perform an upsampling operation to increase the feature image resolution and improve the model accuracy. To further demonstrate the process of lateral transmission of feature information, the output of C1-2 is transmitted to the second ResNet network through a composite connection module as an example (**Figure 8**) C1-2 is the second stage of the first ResNet network, so its output feature map size is 128×128×256. The number of channels of the feature map is downsampled by CC1 with a 1×1×64 convolution, and the size of theoutput feature map is 128×128×64. In addition, to perform linear summation with the output feature map of C2-1, the length and width parameters of the feature map are further upsampled. Then, the size of the output feature map of CC1 is 256×256×64, which is the same as the size of the output feature map of C2-1.

To obtain the RPN structure and the feature maps that are required for the ROI prediction branches, P2~P5 of the output of the last ResNet network are input into the FPN.

### 2.5 Performance evaluation metrics

In this study, precision rate (P), recall rate (R), and precision-recall curve (PR) are used as evaluation metrics. Precision refers to the prediction result, which is defined as the number of targets that are true positive examples among the targets predicted as positive examples. Recall refers to the sample data, which is defined as the number of targets that are predicted as positive examples among the targets predicted as positive samples. In equations 2 and 3, TP denotes the number of positive samples with correct predictions, FP denotes the number of positive samples with incorrect predictions, and FN denotes the number of negative samples with incorrect predictions. The PR curves allow us to analyze the dynamic trends of the accuracy rate and recall rate on the whole data set and compare the performance of different models on the same data set. The calculation formulas are as follows:


(2) $$P=\frac{TP}{TP+FP}$$

(3) $$R=\frac{TP}{TP+FN}$$

AP summarizes the shape of PR curve from the numerical level, and its value is the average of accuracy at the recall level with equal intervals of 0 to 1. The calculation formula is:


(4) $$AP(c)=\int_{0}^{1}Precision\;rate(c)\;dRecall\;rate(c)$$

where C represents the goal of a category. In order to evaluate the effectiveness of the proposed Citrus green fruit detection method, the precision-recall curve and average accuracy AP50 and AP75 are used as evaluation indexes. AP50 and AP75 represent the average accuracy when IoU threshold is set as 50 and 75.

### 2.6 Model training and testing

The computer hardware configuration parameters that are used in this test are as follows: The operating system is Ubuntu18.04, the processor is an Intel Xeon(R) CPU E5-2620 V4 @ 2.1 GHz ×16, and the memory is 64 GB. The graphics processing unit (GPU) is a GTX TIAN X.

Migration learning (Shilei et al., 2019) is a machine learning method for knowledge domain migration. The core strategy is as follows: Knowledge models of mature domains, which are obtained by algorithms learning on massive data for a long time, are applied to the model training of new domains, where the similarity between data and domains is used to share parameters with models of new domains to reduce hardware resource consumption, while migration learning builds rich low-level semantic featuresfor the models and guides the models to learn appropriate weighting parameters parameters (Yin et al., 2020). The citrus green fruit dataset that is collected in this study is obviously insufficient to support the data size that is required for learning the parameters from no initial information.

Based on the above strategy, this study utilizes the pretraining weights of the Mask-RCNN algorithm on the COCO dataset to pretrain the model in the initial stage of model training and then fine-tunes the model using the citrus green fruit dataset that isestablished in this study (Wang and Xiao, 2021). The training is backpropagated using minibatch gradient descent (MBGD) to optimally update the model parameters. During training, the batch size is set to 2, and the network model initialization learning rate is set to 0.001. It lasts for 30 epochs in total. After training, the loss values of the model are recorded after each iteration, and the correlation curves between the number of iterations and the loss values are plotted to analyze the accuracy change and convergence of the model during training. The final converged model is saved, and then model prediction is performed on the test set. The average precision, accuracy, and recall are calculated and saved, and the PR curve is plotted to analyze the generalization ability of the model.

## 3 Results

### 3.1 Performance comparison of different algorithms

To evaluate the effectiveness of the data enhancement method in solving the overfitting problem, the Mask-RCNN algorithm is used for training and testing on the citrus green fruit images before and after data enhancement. After training on 557 original images, the model has an AP value of 71.31% on 446 test set images, and after training on 1782 images with data enhancement, the model has an AP value of 92.47% on 446 test set images, as presented in **Table 1**.

**Table 1:** Data enhancement test results.

Models	AP
Mask-RCNN (original)	71.31
**Mask-RCNN (enhanced)**	**92.47**

AP: Average Precision.

Bold values represent the best indicators in the current table.

Based on the data-enhanced citrus green fruit dataset, the Mask-RCNN algorithm integrated with CB-Net is compared with the traditional Mask-RCNN algorithm. ResNet50 and ResNet101 are selected as the feature extraction backbone networks. Four groups of experiments are conducted, namely, Mask-RCNN+ResNet50, Mask-RCNN+ResNet101, Improved Mask-RCNN+ResNet50, and improved Mask-RCNN+ResNet101.

**Figure 9:**
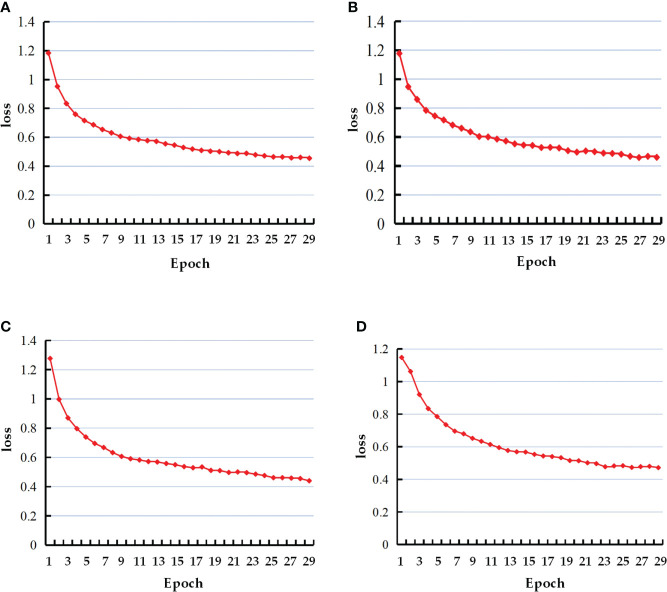
Loss curves of the four models. **(A)** Mask-RCNN+ResNet50; **(B)** Mask-RCNN+ResNet101; **(C)** Improved Mask-RCNN+ResNet50; **(D)** Improved Mask-RCNN+ResNet50.

The loss value variance curves of the four groups of tests are shown in **Figure 9**. The loss values of all four models eventually converge between 0.4 and 0.5, and the four models reach better values within 30 training iterations.

### 3.2 Comparison of improved model with other models

**Figure 10:**
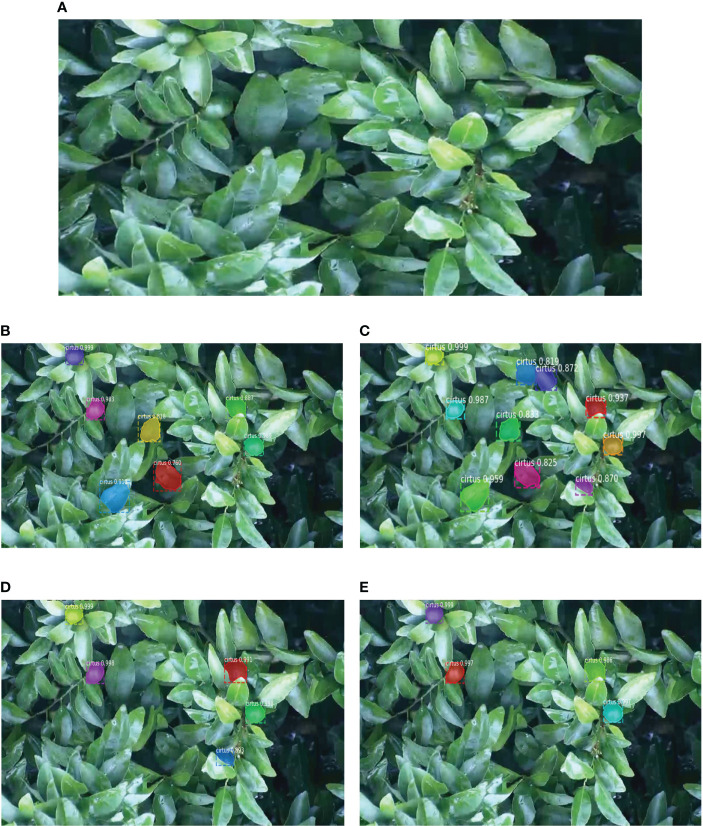
Forecast visualization results of the four models. **(A)** Original image; **(B)** Mask-RCNN+ResNet50 model; **(C)** Mask-RCNN+ResNet101 model; **(D)** Improved Mask-RCNN+ResNet50 model; **(E)** Improved Mask-RCNN+ResNet101 model.

To evaluate the performance of the proposed model, training is performed on the citrus green fruit dataset that is constructed in this study, and the improvement points are compared one by one. The results are presented in **Table 2**. The average accuracy of the improved Mask-RCNN+ResNet50 model on the citrus green fruit dataset that is built in this study is 95.36, which is 1.42%, 3.13%, and 2.17% better than those of the other three models. The results from the segmentation prediction are visualized in **Figure 10**. The number of real citrus green fruits in the original image is 5. The improved Mask-RCNN+ResNet50 model correctly and completely identifies all citrus green fruits. The other three models misjudge the green leaves as citrus green fruits or miss the detection of citrus green fruits. Among them, the Mask-RCNN+ResNet50 model misjudges 3 and misses 1, the Mask-RCNN+ResNet101 model misjudges 5, and the improved Mask-RCNN+ResNet101 model misses 1. The results show that the improvedMask-RCNN+ResNet50 model greatly improves the accuracy of the detection and segmentation of citrus green fruit.

**Table 2:** Prediction results of four sets of trials.

Models	AP_50_	AP_75_
Mask-RCNN+ResNet50	94.03	92.17
Mask-RCNN+ResNet101	92.47	89.87
**Improved Mask-RCNN+ResNet50**	**95.36**	**93.45**
Improved Mask-RCNN+ResNet101	93.34	91.68

AP_50_: The IOU threshold is 0.5.

AP_75_: The IOU threshold is 0.75.

Bold values represent the best indicators in the current table.

**Figure 11:**
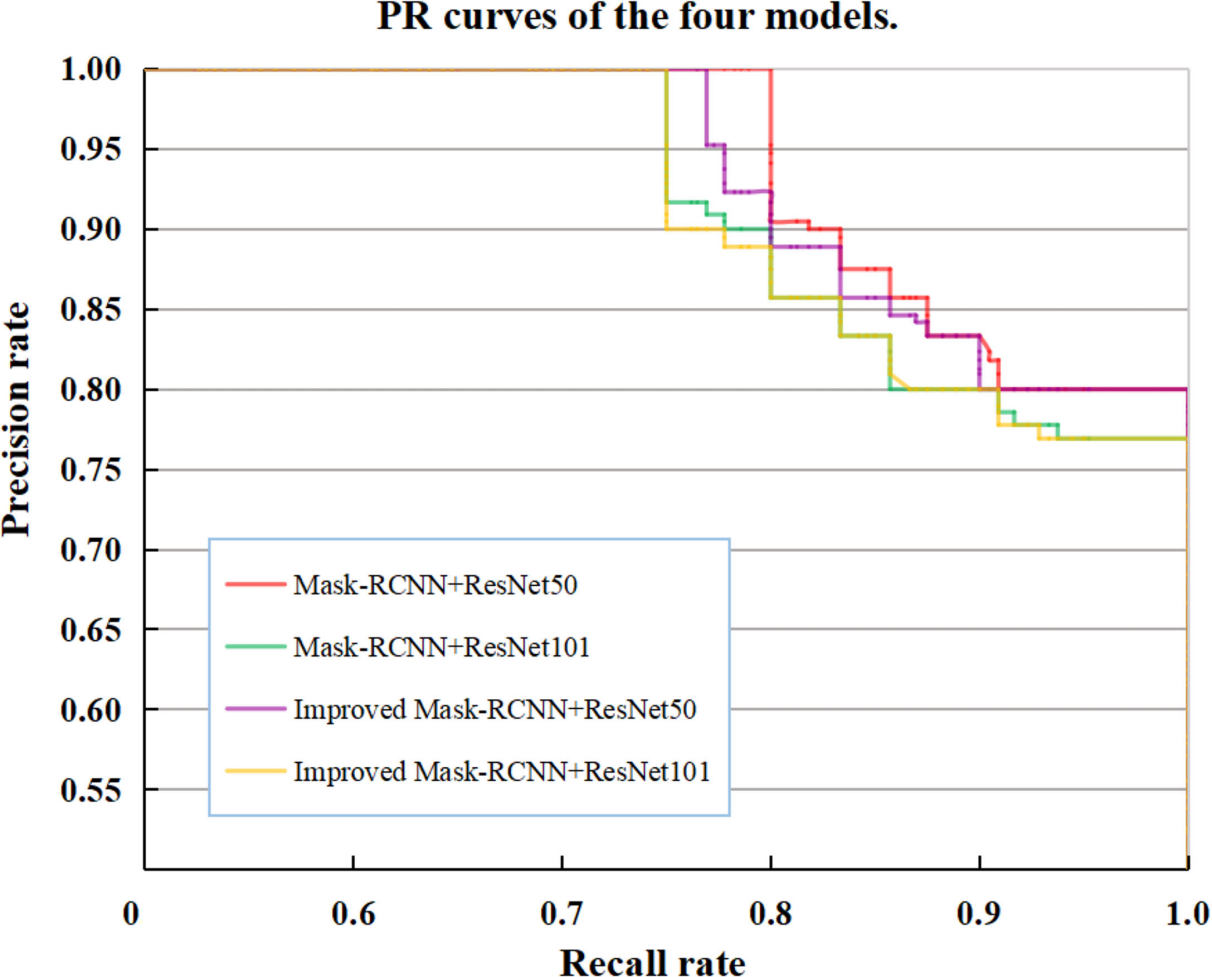
PR curves of the four models.

The two-stage target detection algorithm usually first uses the algorithm (selective search or region proposal network, etc.) to extract candidate frames from the image, and then performs secondary correction on the candidate frame target to obtain the detection result. Therefore, the accuracy of candidate frame selection is particularly important for the target detection task. In the feature extraction stage, this study mainly uses the adoption of FPN to enhance the expression of multi-scale features of citrus green fruits. In a further step, the feature map is passed through the RPN and ROI Align modules to generate and correct candidate frames. In order to verify the accuracy of the candidate frames of the improved model, we calculate the accuracy and recall rates of the four groups of experiments on the test set and plot the PR curves, as shown in **Figure 11**. The area that is enclosed by each PR curve and the coordinate axes reflects the accuracy of the candidate frame. When the area is larger, the confidence of the candidate bound that is predicted by the model is higher. The areas that are enclosed by the PR curve and the coordinate axes of the four models are calculated, and the results are presented in **Table 3**. The area that is enclosed by the PR curve and the coordinate axes of the improved Mask-RCNN+ResNet50 model is 0.9673, which is 0.3%, 1.3%, and 1.2% larger than the areas of the other three models.

**Table 3:** Areas that are enclosed by the PR curves and coordinate axes of the four models.

Models	Areas
Mask-RCNN+ResNet50	0.9638
Mask-RCNN+ResNet101	0.9549
**Improved Mask-RCNN+ResNet50**	**0.9673**
Improved Mask-RCNN+ResNet101	0.9556

Bold values represent the best indicators in the current table.

## 4 Application case study

**Figure 12:**
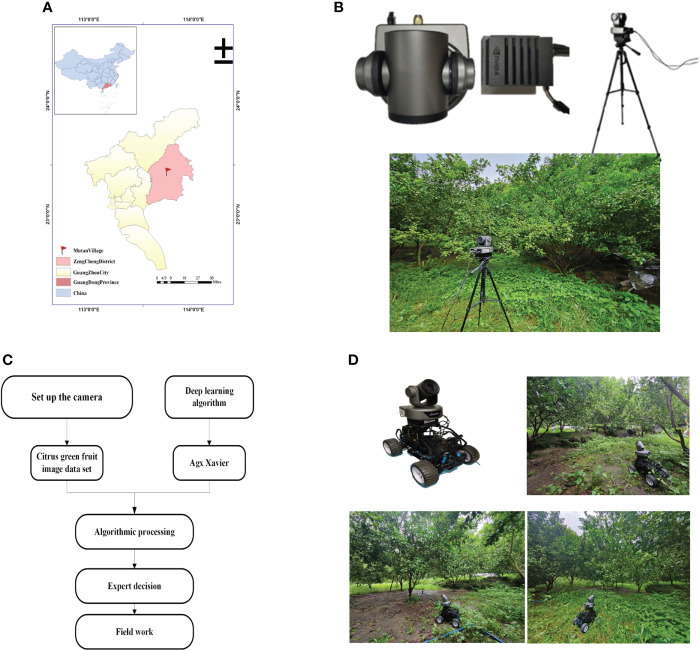
**(A)**Example application location; **(B)** HD camera, Agx Xavier, Overall build, Field layout effect;**(C)** Overall flow chart; **(D)** Unmanned vehicle and unmanned vehicle for citrus green fruit dynamic detection.

Citrus green fruit thinning assisted identification detection was conducted in the citrus orchard using the improved model that was suggested in this paper and transplanted to NVIDIA Agx Xavier. The citrus orchard is located in Mutan Village, Zengcheng City, Guangdong Province, China (as shown in **Figure 12A**). The specific parameters of the equipment used are as follows: NVIDIA Agx Xavier, the processor model is 8-core ARM v8.2, the memory model is eMMC5.1, the size is 32GB, and the GPU (Graphics Processing Unit) is 512-core Volta GPU with Tensor Cores. High-definition camera (Daipu DP-UK100), the size of HDCMOS sensor is 1/2.8 inches, the effective pixel is 2.1 million, and it can shoot 1080P ultra-high-resolution images.

Before deploying the model with an edge smart station, it is necessary to initialize the edge smart station. In order to facilitate subsequent debugging and development, the Jetpack component library is used to reinstall the Agx Xavier system. The reinstalled operating system is Ubuntu 18.04, with GPU acceleration application tools such as CUDA 10.2.89 and Cudnn 8.0.0.180, and visual computing tools such as OpenCV and Vision Works. On this basis, according to the CUDA version, install thedeep learning environment required by Mask RCNN. The environment is as follows: Tensorflow gpu 1.15.0, keras 2.1.3, scikit image. Then, we connect the high-definition camera to the edge smart station through the USB interface, and configure the camera interface. We use the OpenCV toolkit to obtain the real-time image data of the camera, and call the model to detect each image acquired in real time.

Place Agx Xavier and the camera on a tripod at a distance of 2-3 meters from the citrus canopy and 1.6 meters above the ground (as shown in **Figure 12B**). A total of 10 collection points were set up in the orchard, and the focal length and angle of the camera were adjusted according to the position of the equipment and the green fruit. The camera took 2 hours each time. Both data collection and model reasoning were processed on Agx Xavier, and the processed results would be analyzed by the systemto further give fruit thinning decision-making suggestions. The overall flow chart is shown in **Figure 12C**. After the improved model was transplanted to Agx Xavier, the recognition of green fruits was in line with the performance indexes achieved during the model training test, and the inference time was 0.939 seconds, which could satisfy the demand for automatic green fruit recognition in citrus orchard thinning operations.

The equipment need to be manually moved about to collect various tree plants because it is mounted on a fixed tripod with a small field of view. In order to expand the applicability of the system in the future and further increase the robustness and feasibility of the system, the improved model proposed in this paper can be deployed on an unmanned vehicle in the future (as shown in **Figure 12D**). The method is as follows, an NVIDIA Agx Xavier that has been ported with an improved model and a HD camera is built on the unmanned vehicle. Then, by manually setting fixed trajectories in citrus orchards in advance, the unmanned vehicle will move along the preset trajectories when performing the task of citrus green fruit yield detection in citrus orchards. During the task, the following operations are repeated: (1) the unmanned vehicle is stationary once every 2 minutes (2) the HD camera takes pictures to obtain real-time image data (3) Agx Xavier invokes the model to detect each image obtained in real time. This enables the acquisition of image data of the whole orchard with low labor cost.

## 5 Discussion

To address the problem of difficult identification of citrus green fruits in the natural environment, an instance segmentation method based on the strategy of feature fusion is proposed for reducing the influence between citrus green fruit features and irrelevant features, and we construct a novel Mask-RCNN model using CB-Net (a composite backbone network). The generalization ability of the neural network was improved by constructing the green citrus dataset and a series of dataset preprocessing operations. Second, a combined connection block is introduced to reduce the number of channels and improve the model accuracy. The accuracy of the final trained model on the test set is 95.36%, which is higher than that of Mask-RCNN. This indicates that the improved model can more accurately identify more citrus green fruits.

To further verify the effectiveness and feasibility of this method, the performance of the proposed model in detecting different individuals under the same class of target to obtain the morphology of citrus green fruits in advance is evaluated. Compared with the original method, the average detection accuracy of the improved Mask-RCNN model is 95.36%, increased by 1.42%, and the area surrounded by precision-recall curve and coordinate axis is 0.9673, increased by 0.3%. The loss values of all models eventually converged between 0.4 and 0.5. The results show that compared with Mask-RCNN model, data augmentation can solve the model overfitting problem that is caused by the small amount of data. By fusing the multi-layer features of Citrus green fruits, the improved model can pay more attention to the obvious regions and more detailed features in each image, so as to improve the accuracy of citrus green fruit detection and segmentation.

Under the conditions of this study, the loss value of the ResNet50 network converged slightly faster than that of the ResNet101 network, and the model accuracy of the ResNet50 network was 1.93% higher than that of the ResNet101 network. The reason for this phenomenon is that the parameter scale of ResNet101 is larger than that of ResNet50, and the loss value is related to the update speed of the weight parameters. Compared with the multicategory and multi scene target detection task, the semantic information of the citrus green fruit images that were collected in this study is not obvious, and the low level of demand for rich semantic features that are generated at higher stages results in data-level limitations,the feature maps with too many semantic features results in model overfitting. Therefore, the improved Mask-RCNN +ResNet50 model had better inference results on the test set, thereby indicating that the model had more accurate fitting and better generalization ability for single citrus green fruit characteristics.

Based on the improved model proposed in this study, we carried out the assisted recognizing detection of citrus green fruit thinning. The results show that when the improved model proposed in this paper is applied in the field, the accuracy meets the training effect, and the average inference time of the system is 0.969s, which meets the requirements of real-time detection. The improved model proposed in this paper has good generalization ability and can be practically applied to the identification of citrus green fruits in citrus orchards. The inference results can further help the development of fruit thinning in orchards.

Based on the observations of this study, the designed model has various limitations. In a more complex citrus orchard scene, the overlap effect cannot be ignored when the number of recognized citrus green fruits is large and the pixel sizes do not differ much. In the next step of research, we need to further differentiate the analysis and training for different scenes, and the model will be lightly modified to further improve its generalization ability to enhance its detection accuracy and speed.

## 6 Conclusions

Using computer vision technologies to establish a citrus detection model and realize the real-time processing of orchard images through sensor systems and high-performance computers is an important development trend of smart agriculture in citrus detection at present. This study designed a citrus green fruit detection method based on improved feature network extraction. Citrus green fruits were selected as the research objects. To ensure the accurate detection of different individuals of the same target category in the collected images in the actual detection and recognition process, based on the Mask-RCNN algorithm, CB-Net with deep and shallow fusion was innovatively combined with the traditional feature extraction network ResNet to fuse the multilayer features of citrus green fruits. Then, the proposed model was compared with the traditional model in detail.

The improved model that was proposed in this study showed an average accuracy of 95.36 on the citrus green fruit dataset that was established in this study, and the accuracy of the model increased by 1.18% after the same feature extraction network was integrated into CB-Net. The improved model has higher ability to express the characteristics of citrus green fruits and reduces the interference of complex image backgrounds in citrus green fruit instance segmentation, and the detection accuracy has exceeded the basic accuracy requirements. Compared with the current common algorithms, the improved model has accuracy advantages and edge erection feasibility. In a data enhancement contrast test, it was verified that the data enhancement method that was proposed in this study can solve the overfitting problem that is caused by the limited amount of data. The results showed that the proposed algorithm based on the improved Mask-RCNN has improved citrus green fruit detection accuracy and speed. A performance comparison of the Mask-RCNN algorithm before and after the improvement showed that the optimal model for citrus green fruit detection and segmentation is the improved Mask-RCNN+ResNet50 model. The results showed that the ResNet50 network with low-level semantic information performed better on the dataset in this study. CB-Net promotes the extraction and fusion of features and further improves the generalization ability of the model to unknown data.

In the future, the citrus green fruit detection and segmentation model can be further applied to an edge intelligent platform with high computing power. The deployment of the model at the edge is another important strategy for citrus orchard data transmission. It can provide technical support for the management and control system of the citrus key growth period and provide a reference for the realization of scientific and unmanned farm construction.

## Data availability statement

The raw data supporting the conclusions of this article will be made available by the authors, without undue reservation.

## Author contributions

JLu conceptualized the experiments, selected the algorithms, collected and analyzed the data. RY, CY and JLi wrote the manuscript. HW and WC trained the algorithms and collected and analyzed the data. XC, WW and YL supervised the project and revised the manuscript. All authors discussed and revised the manuscript. All authors contributed to the article and approved the submitted version.

## Funding

This work was supported by the Laboratory of Lingnan Modern Agriculture Project (NT2021009), Basic and Applied Basic Research Project of Guangzhou Basic Research Plan in 2022 (202201010077), The 111 Project (D18019), Guangzhou Key R&D project (SL2022B03J01345), The Open Research Fund of Guangdong Key Laboratory for New Technology Research of Vegetables (201704) and Guangdong Province Enterprise Science and Technology Special Ombudsman Project (GDKTP2020070200).

## Conflict of interest

The authors declare that the research was conducted in the absence of any commercial or financial relationships that could be construed as a potential conflict of interest.

## Publisher’s note

All claims expressed in this article are solely those of the authors and do not necessarily represent those of their affiliated organizations, or those of the publisher, the editors and the reviewers. Any product that may be evaluated in this article, or claim that may be made by its manufacturer, is not guaranteed or endorsed by the publisher.

## Abbreviations

Mask-RCNN, Regions with Convolutional Neural Network features; Res4b, A module in the Residual network; ResNet, Residual network; CB-Net, Composite Backbone Network; COCO, Microsoft Common Objects in Context; HLB, HuangLongBing disease; RPN, Region Proposal Network; Faster-RCNN, Faster Region Convolutional Neural Networks; VGG16, Visual Geometry Group Network16; CNN, Convolutional Neural Network; FPN, Feature Pyramid Network; RoI, Region of Interest; CC, composite connection; AHLC, Adjacent high-level composition; AP, Average Precision; IoU, Intersection over Union.
